# ENGAGING AFRICAN AMERICAN FAMILY CAREGIVERS THROUGH ART AND CREATIVE ACTIVITIES: A CASE STUDY

**DOI:** 10.1093/geroni/igac059.2058

**Published:** 2022-12-20

**Authors:** Afeez Hazzan, Luvon Sheppard, Joyce Hazzan

**Affiliations:** State University of New York at Brockport, Hilton, New York, United States; Rochester Institute of Technology, Rochester, New York, United States; Liberty University, Lynchburg, Virginia, United States

## Abstract

Studies have shown that engaging family caregivers of people with dementia in creative activities can provide therapeutic benefits by relieving stress and promoting well-being. However, there is a dearth of studies focusing on the involvement of racial minority family caregivers of people with dementia in research involving art and creative activities. The purpose of this study is to present the results of a case study interview with an artist-educator-community collaborator who acted as a key facilitator for the ACTION ARTS study. The purpose of the ACTION ARTS study was to utilize mobile technology applications for facilitating creative activities to enhance health and well-being of African-American family caregivers and their loved ones in the middle-to-late stages of dementia. The study included activities such as memory stimulation, art viewing, art making, and other forms of creative expressions. The research question for the current case study was: What are the best practices for engaging African-American family caregivers of older adults in the middle-to-late stages of dementia in research involving art and creative activities? Thematic analysis of the qualitative data from the case study interview yielded the following recommendations/results (1) Design the program of art and/or creative activities in an easy way to facilitate interaction among participants; (2) Make the program as accessible and relevant as possible so everyone is able to relate to it; (3) Emphasize areas of commonality that could help in giving both the facilitators and participants food for thought.

